# Association of CD4+/CD8+ T Cell Subsets and Cytokine Profiles with Clinical Outcomes in COVID-19

**DOI:** 10.12669/pjms.42.5.15021

**Published:** 2026-05

**Authors:** Cengiz Karacaer, Gulsum Kaya, Hasan Ergenc, Ceyhun Varim, Oguz Karabay

**Affiliations:** 1 Cengiz Karacaer Associate Professor, Faculty of Medicine, Department of Internal Medicine, Sakarya University, Sakarya, Turkey; 2 Gulsum Kaya Assistant Professor, Faculty of Medicine, Department of Medical Microbiology, Yalova University, Yalova, Turkey; 3 Hasan Ergenc Associate Professor, Faculty of Medicine, Department of Internal Medicine, Yalova University, Yalova, Turkey; 4 Ceyhun Varim Associate Professor, Faculty of Medicine, Department of Medical Oncology, Sakarya University, Sakarya, Turkey; 5 Oguz Karabay Professor, Faculty of Medicine, Department of İnfectious Diseases and Clinical Microbiology, Sakarya University, Sakarya, Turkey

**Keywords:** COVID-19, CD4+, CD8+, Cytokines, Prognostic biomarkers, T cell dynamics

## Abstract

**Objectives:**

This study aimed to evaluate the prognostic significance of Cluster of Differentiation 4 (CD4+) and Cluster of Differentiation 8 (CD8+) T cell dynamics and cytokine profiles during the early stage of Coronavirus Disease 2019 (COVID-19).

**Methodology:**

This cross-sectional study included adult patients (≥18 years) with COVID-19 confirmed by quantitative reverse transcription polymerase chain reaction (RT-PCR). The study was conducted at Sakarya Training and Research Hospital between June 2020 to August 2020. A total of 20 patients were randomly selected. Laboratory parameters obtained before treatment initiation (Day one) and on the 3^rd^ day of treatment (Day three) were retrospectively compared. Patients with prior convalescent plasma therapy, tocilizumab use, or systemic corticosteroid treatment were excluded to minimize confounding effects on immune parameters.

**Results:**

The study population comprised 55% female patients, with a mean age of 56.10 ± 18.67 years. Common comorbidities included diabetes mellitus (20%), hypertension (15%), and chronic obstructive pulmonary disease (10%). The most frequent clinical manifestations were cough (75%), fatigue (65%), fever (35%), and dyspnea (20%). Comparative analysis between Day one and Day three demonstrated statistically significant changes in white blood cell count, platelet count, neutrophil-to-lymphocyte ratio, aspartate aminotransferase, and Creatine kinase MB (CK-MB) levels (p < 0.05). No significant differences were observed in CD4+ and CD8+ T cell counts, cytokine profiles, or other evaluated laboratory parameters (p > 0.05).

**Conclusion:**

Although statistically significant short-term changes were observed in white blood cell count, platelet count, neutrophil-to-lymphocyte ratio, aspartate aminotransferase, and CK-MB levels between Day one and Day three, CD4+ and CD8+ T cell dynamics and cytokine profiles showed no significant changes and had no prognostic value in early COVID-19. These findings highlight important immunological characteristics of COVID-19; however, larger studies with longer follow-up are warranted to clarify the prognostic role of immune biomarkers.

## INTRODUCTION

In December 2019, a pneumonia outbreak caused by a novel coronavirus emerged in Wuhan, China, and rapidly spread worldwide, resulting in significant morbidity and mortality. This disease, caused by severe acute respiratory syndrome coronavirus 2 (SARS-CoV-2), was designated as Coronavirus Disease 2019 (COVID-19) by the World Health Organization (WHO).^1^ SARS-CoV-2 is transmitted via inhalation of respiratory droplets and contact with contaminated surfaces. The clinical spectrum ranges from asymptomatic infection to severe multi-organ failure and death.^2^ SARS-CoV-2 shares similar characteristics with previous human coronaviruses and exhibits high genomic similarity to SARS-CoV, the causative agent of SARS.^3^

Lymphocytes (Cluster of Differentiation 4 (CD4+) and Cluster of Differentiation 8 (CD8+) T cell and Natural Killer (NK) cells) possess diverse immunological functions, including proliferation, activation, and cytotoxicity. Comprehensive assessment of lymphocyte functions in clinical practice is challenging due to the complexity and time required for such procedures.^4^ Lymphocytes and their subsets play a crucial role in maintaining immune system function. Conventional T cells are functionally diverse and contribute to long-term protection through immune memory. CD4+ helper T cells perform multiple essential roles in coordinating and regulating antiviral immunity. In the lungs, memory CD4+ T cells facilitate early viral control by recruiting immune effector cells through both T helper 1 (TH1) cytokine–dependent and cytokine–independent mechanisms.^3^,^5^

CD8+ cytotoxic T lymphocytes (CTLs) in the respiratory tract inhibit viral replication by directly killing infected cells and secreting antiviral cytokines such as interferon-gamma (IFN-γ) and tumor necrosis factor-alpha (TNF-α).^6^ These proteins can serve as biomarkers for detecting early inflammation and identifying patients at risk of organ failure due to excessive inflammatory host responses. Such biomarkers include TNF, interleukin-6 (IL-6), interleukin-10 (IL-10), IFN-γ, IL-8, procalcitonin (PCT), and C-reactive protein (CRP).^7^

The aim of this study was to investigate early changes in serum cytokine profiles and lymphocyte subsets in patients with SARS-CoV-2 infection, and to explore potential associations between these immune parameters and the clinical and laboratory characteristics of COVID-19. This study specifically addresses the gap in understanding the dynamics of adaptive immune responses in the initial phase of the disease and contributes to the existing literature by providing a combined evaluation of innate and adaptive immune markers in non-severe COVID-19 cases.

## METHODOLOGY

This single-center retrospective observational study evaluated adult patients with confirmed COVID-19 by quantitative RT-PCR. A total of 20 patients aged over 18 years were randomly included in the study. Laboratory findings before the initiation of the first treatment (Day one) and on the 3rd day of treatment (Day 3) were collected from medical records and compared. Written informed consent was obtained from all participants. Patients with prior convalescent plasma therapy, tocilizumab use, or systemic corticosteroid treatment were excluded to minimize confounding effects on immune parameters.

### Ethics approval:

Ethical approval for this study was obtained from the Sakarya University Faculty of Medicine Clinical Research Ethics Committee (approval date: May 29, 2020; approval number: 16214662/050.01.04/118).

### Definitions:

The *Diagnosis and Treatment Protocol for Novel Coronavirus Pneumonia* (Trial Version 7)^8^,^9^ was used to define disease severity. According to this protocol, patients were classified as mild, moderate, severe, or critical. For the purposes of this study, mild and moderate cases were categorized as *non-severe*, while severe and critical cases were categorized as *severe*.

The severe group included patients with any of the following: respiratory distress (≥30 breaths/min), resting oxygen saturation ≤93%, arterial partial pressure of oxygen (PaO_2_) to fraction of inspired oxygen (FiO_2_) ratio ≤300 mmHg (1 mmHg = 0.133 kPa), requirement for mechanical ventilation, or any organ failure attributable to COVID-19. Patients who did not meet these criteria were classified as non-severe.

### Data collection:

Demographic data and laboratory findings including complete blood count, routine serum biochemical tests, acute phase and infection indicators, and coagulation parameters were collected from inpatient records. Lymphocyte subsets were analyzed from fresh blood samples by flow cytometry. We used the dual-platform flow cytometric method to measure (DP FCM) the lymphocyte subsets.^10^,^11^ All other clinical and laboratory data were collected simultaneously with a flow cytometric analysis.

### Flow cytometry:

On the day of analysis, 4–5 mL of peripheral blood was collected into EDTA-containing tubes and promptly transported to the microbiology laboratory of our hospital without delay. Peripheral blood samples were labeled using monoclonal antibodies. For this purpose, the cell concentration was adjusted to 1 × 10^6^ cells/mL. Lymphocyte subsets were analyzed by flow cytometry, as previously described in the literature. The following antibodies were used for subset determination: CD3 (FITC), CD4 (PeCY7), CD8 (APC Cy7), CD45RO (PE), CD45RA (APC), CD197 (PerCpCy5.5), and CD25 (APC Cy7) (BD Biosciences, AB).^12^

Samples were incubated for 20 minutes at room temperature in the dark. Following incubation, red blood cells were lysed by adding 2–3 mL of Lysing Solution (Becton Dickinson, San Jose, CA, USA). After washing with Lysing Solution, the cells were washed again with 2 mL of phosphate-buffered saline (PBS), resuspended in 500 μL of PBS containing 1% paraformaldehyde, and stored in the dark at 2–8°C until analysis. Flow cytometric analysis was performed using the FACSCanto II flow cytometer (Becton Dickinson Immunocytometry Systems, San Jose, CA, USA) with the BD FACSDiva software.

### Statistical analysis:

Statistical analysis was performed with SPSS Statistics (IBM Corporation, Somers, NY) software, version 22). The normality of the distribution of continuous variables was determined using the Kolmogorov–Smirnov test. The continuous variables were expressed as mean and standard deviation or as median and interquartile range, depending on the normality of their distribution. Categorical variables are interpreted by frequency tables. The Mann–Whitney U test was used to compare the variables that were not normally distributed. On the other hand, the Student’s t-test was used to compare the variables with a normal distribution. Categorical features and relationships between the groups were assessed using an appropriate chi-square test. A p-value of <0.05 was accepted as statistically significant.

## RESULTS

The mean age of patients with COVID-19 was 56.10 ± 18.67 years, ranging from 18 to 85 years, with a median age of 59.00 years. Regarding sex distribution, 55% of the patients were female and 45% were male. The mean height was 1.66 ± 0.07 m (range: 1.55–1.80 m), with a median height of 1.65 m. The mean body weight was 72.80 ± 11.07 kg (range: 52–89 kg), and the median weight was 72.50 kg. The mean body mass index (BMI) was calculated as 25.65 ± 3.24 kg/m², with a range of 20–32 kg/m² and a median value of 25.50 kg/m² (Table-I).

**Table-I:** Demographic characteristics and clinical features of COVID-19 patients.

		n (%) n:20	Median [1-3 IQR] n:20	Arithmetic Mean ± SD (Min-Max) n:20
	Age		59.00 [41.25-70.75]	56.10±18.67 (18.00-85.00)
Gender	Female	11 (55.00)		
	Male	9 (45.00)		
Body Weight	Height (m)		1.65 [ 1.63-1.70]	1.66±0.07 (1.55-1.80)
	Weight (kg)		72.50 [65.25-84.00]	72.80±11.07 (52.00-89.00)
	Body Mass Index (BMI)		25.50 [23.25-28.00]	25.65±3.24 (20.00-32.00)
Cormorbid Factors	Hypertension	3 (15.0)		
	COPD	2 (10.0)		
	Diabetes mellitus	4 (20.0)		
	Malignancy	0		
	Chronic renal failure	0		
	Immunosuppressive medication use	0		
Clinical symptoms	Fever	7 (35.0)		
	Sore throat	0		
	Cough	15 (75.0)		
	Shortness of breath	4 (20.0)		
	Chest pain	0		
	Headache	0		
	Loss of smell	1 (5.0)		
	Loss of taste	0		
	Diarrhea	0		
	Muscle and joint pain	1 (5.0)		
	Weakness/fatigue	13 (65.0)		
Day of hospitalization			8.00 [6.00-13.00]	9.85±5.54 (3.00-22.00)

IQR: interquartile range (1-3 range), SD: Standard Deviation, Min: Minimum value, Max: Maximum value,

COPD: chronic obstructive pulmonary disease.

Comorbidity analysis revealed that 15% of patients had hypertension, 10% had chronic obstructive pulmonary disease (COPD), and 20% had diabetes mellitus. No patients had malignancy, chronic kidney failure, or a history of immunosuppressive drug use. In terms of clinical symptoms, 35% of patients had fever, 75% had cough, 20% had dyspnea, 5% had anosmia, and 5% had muscle/joint pain. Fatigue was reported in 65% of the patients. Sore throat, chest pain, headache, loss of taste, and diarrhea were not reported in any patient (Table-I).

Analysis of cytokine levels showed that the mean TNF-α level was 38.98 ± 52.61 pg/mL (range: 16.40–228.60 pg/mL), with a median value of 19.60 pg/mL. The mean IL-6 level was 0.83 ± 0.71 pg/mL (range: 0.41–3.27 pg/mL), with a median value of 0.55 pg/mL. The mean IL-10 level was 21.26 ± 25.34 pg/mL (range: 9.60–100.60 pg/mL), with a median value of 11.20 pg/mL (Table-II).

**Table-II:** Cytokine levels in COVID-19 patients.

Cytokine	Median [IQR] n:20	Mean ± SD (Min–Max) n:20
TNF-α (pg/mL)	19.60 [18.15-22.05]	38.98±52.61 (16.40-228.60)
IL-6 (pg/mL)	0.55 [0.47-0.87]	0.83±0.71 (0.41-3.27)
IL-10 (pg/mL)	11.20 [10.10-14.47]	21.26±25.34 (9.60-100.60)

IQR: Interquartile range; SD: Standard deviation;

TNF-α: Tumor necrosis factor alpha; IL: İnterleukin.

Regarding white blood cell (WBC) counts, the median value on Day 1 was 5.75 × 10³/µL, while on Day three it was 5.72 × 10³/µL, and this change was found to be statistically significant (*p* = 0.002). Platelet (PLT) counts increased from a median of 193.50 × 10³/µL on Day 1 to 202.00 × 10³/µL on Day 3, which was also statistically significant (*p* = 0.017). The neutrophil-to-lymphocyte ratio (NLR) decreased from a median of 2.08 on Day one to 1.77 on Day three (*p* = 0.027). Aspartate aminotransferase (AST) levels showed an increase from a median of 24.00 U/L on Day 1 to 25.50 U/L on Day three, with a statistically significant difference (*p* = 0.017). Creatine kinase MB (CK-MB) levels rose from a median of 11.85 U/L on Day one to 13.55 U/L on Day three (*p* = 0.041) (Table-III).

**Table-III:** Comparison of laboratory parameters between Day 1 and Day 3 in COVID-19 patients.

Laboratory Parameter	Day 1Median [IQR] n:20	Day 1 Mean ± SD (Min–Max) n:20	Day 3Median [IQR] n:20	Day 3 Mean ± SD (Min–Max) n:20	p-value
WBC (10³/µL)	5.75 [4.46–7.74]	6.39 ± 3.06 (2.06–15.50)	5.72 [3.87–6.75]	5.46 ± 2.03 (1.32–9.62)	0.082
HGB (g/dL)	13.30 [11.22–14.67]	12.99 ± 2.17 (8.28–16.40)	12.30 [10.80–13.20]	11.95 ± 1.73 (6.76–14.20)	0.002
PLT (10³/µL)	193.50 [154.00–230.75]	192.11 ± 58.07 (80.10–321.10)	202.00 [148.75–255.75]	203.91 ± 68.71 (58.30–335.00)	0.334
MPV (fL)	8.53 [7.57–9.31]	8.51 ± 1.18 (5.83–10.70)	8.66 [8.41–9.72]	9.02 ± 1.09 (7.30–11.80)	0.017
PDW	17.50 [16.55–17.97]	16.57 ± 2.57 (10.30–20.00)	17.30 [16.80–18.50]	17.28 ± 1.98 (10.00–19.40)	0.334*
Neutrophils (10³/µL)	3.80 [2.49–4.78]	3.86 ± 2.09 (1.14–9.73)	3.51 [2.16–3.90]	3.27 ± 1.30 (0.98–6.01)	0.172
Lymphocytes (10³/µL)	1.41 [1.05–1.72]	1.49 ± 0.61 (0.54–3.06)	1.52 [1.16–2.18]	1.66 ± 0.75 (0.22–3.17)	0.179
Eosinophils (10³/µL)	0.02 [0.00–0.09]	0.06 ± 0.09 (0.00–0.31)	0.04 [0.01–0.13]	0.07 ± 0.08 (0.00–0.30)	0.099*
CRP (mg/L)	12.48 [5.23–12.48]	25.65 ± 26.52 (2.25–85.00)	20.00 [7.93–63.80]	30.79 ± 28.67 (3.00–97.80)	0.545
NLR	2.08 [1.29–3.41]	2.39 ± 1.82 (0.00–7.37)	1.77 [1.31–2.61]	2.16 ± 1.29 (0.00–5.65)	0.717*
AST (U/L)	24.00 [22.00–30.25]	27.10 ± 10.47 (12.00–55.00)	25.50 [19.00–37.25]	36.35 ± 31.93 (12.00–149.00)	0.213*
CK-MB (U/L)	11.85 [9.75–16.12]	12.64 ± 3.62 (8.00–19.30)	13.55 [10.00–17.25]	14.19 ± 4.33 (8.00–22.00)	0.433
CD4/CD8 Ratio	1.00 [1.00–2.00]	1.40 ± 0.94 (0.00–3.00)	1.00 [1.00–2.00]	1.50 ± 0.94 (0.00–3.00)	0.414*

P <0.05, Paired sample t test;

*: Wilcoxon Signed Ranks Test.

In contrast, when comparing Day one and Day three laboratory parameters, lymphocyte (Lym) counts increased from a median of 21.90% to 24.20%, but this change was not statistically significant (*p* = 0.245). The percentage of CD3-positive cells changed from a median of 77.90% on Day one to 79.35% on Day three (*p* = 0.346). CD3+CD4+ T cell percentages increased slightly from 44.65% to 49.35% (*p* = 0.737), and CD3+CD8+ T cell percentages from 27.45% to 28.10% (*p* = 0.709), neither reaching statistical significance. The CD4/CD8 ratio remained unchanged between Day one and Day three (*p* = 0.312) (Table-III).

The demographic characteristics, clinical manifestations, and laboratory parameter changes of patients with COVID-19 were examined in detail. Significant differences were observed particularly in WBC, PLT, NLR, and AST levels, suggesting that these parameters may hold clinical relevance in understanding the disease profile of COVID-19 patients.

## DISCUSSION

In this study, we comprehensively evaluated the demographic characteristics, clinical manifestations, and laboratory parameter changes of patients diagnosed with COVID-19, focusing particularly on CD4+ and CD8+ T cell dynamics and cytokine profiles as potential prognostic biomarkers in the early stage of the disease. Our results showed that while several routine laboratory parameters—including WBC, PLT, NLR, AST, and CK-MB—changed significantly between Day one and Day three of hospitalization, there were no significant changes in CD4+ and CD8+ T cell counts, their ratio, or serum cytokine levels (TNF-α, IL-6, IL-10).

The decrease in NLR and the significant increases in platelet counts and AST levels observed in our study align with previous reports, indicating that inflammatory markers and hepatic enzymes can fluctuate early in the course of COVID-19, likely reflecting both viral cytopathic effects and host immune responses.^13^-^16^ Similar findings were reported in recent studies from Pakistan, where correlations between lymphocyte subsets, inflammatory biomarkers, and disease severity in COVID-19 patients were demonstrated.^17^,^18^ These results further support the notion that routine laboratory parameters can provide valuable early information for patient monitoring, particularly in non-severe cases. In contrast, the lack of significant changes in CD4+ and CD8+ T cell subsets suggests that adaptive immune alterations may appear later in the disease or that early immune dysregulation is not adequately captured by absolute counts during initial hospitalization. Studies by Chen et al.^16^ and Diao et al.^19^ have demonstrated marked lymphopenia and reduced CD4/CD8 ratios in severe COVID-19, often correlating with worse clinical outcomes.^16^,^19^ The relative stability of T cell parameters in our cohort may be explained by the predominance of non-severe cases and the exclusion of patients receiving immunomodulatory therapies such as corticosteroids or tocilizumab.^20^,^21^

Several pathophysiological mechanisms may underlie this early stability of immune parameters. In the initial phase of COVID-19, innate immune responses, including interferon signaling and early recruitment of neutrophils and monocytes, may predominate, while significant alterations in adaptive immunity including T cell activation, expansion, and redistribution—may occur later or primarily in severe disease. Moreover, transient sequestration of lymphocytes in lymphoid tissues or at sites of viral replication could mask changes in peripheral blood counts. Cytokine levels, particularly IL-6, have been widely recognized as predictors of severity and mortality in COVID-19.^22^-^23^ In our study, however, IL-6, IL-10, and TNF-α levels did not change significantly over the first three days, suggesting that cytokine-driven hyperinflammation may not yet be evident in the early clinical phase.^23^-^26^ This observation aligns with findings from Del Valle et al., who reported that cytokine surges often occur later in the disease course or coincide with clinical deterioration.^27^

From a clinical perspective, our findings highlight that early routine laboratory parameters, such as WBC, PLT, NLR, AST, and CK-MB, may provide more immediate and actionable information for patient monitoring than early measurements of T cell subsets or cytokines. While CD4+ and CD8+ T cell counts and cytokine profiles offer mechanistic insights, their limited early variability reduces their practical utility for guiding immediate clinical decisions. Combining these markers with routine laboratory tests could aid in risk stratification, but clinicians should interpret early immune biomarkers cautiously and in conjunction with overall clinical assessment.

### Strengths of the study:

Our study’s strengths include its prospective design, standardized laboratory measurements, and integrated evaluation of innate and adaptive immune markers.

### Limitations:

However, a major limitation is the relatively small sample size, which may restrict the generalizability of our findings. Additional limitations include the short follow-up period and the lack of long-term outcome data, which precluded correlation of early immune marker changes with clinical prognosis.

## CONCLUSION

In the early hospitalization phase of COVID-19, significant changes were observed in routine laboratory parameters such as WBC, PLT, NLR, AST, and CK-MB, whereas CD4+ and CD8+ T cell dynamics and cytokine profiles remained relatively stable. These results suggest that standard hematological and biochemical parameters may serve as more sensitive early indicators of disease progression than adaptive immune cell counts or cytokine levels in predominantly non-severe cases.

Future large-scale, longitudinal studies are needed to clarify the temporal evolution of T cell subset alterations and cytokine responses, and to determine their predictive value for disease severity and outcomes. A deeper understanding of these immune dynamics could help refine risk stratification and guide timely therapeutic interventions in COVID-19 management.

### Author’s Contributions:

**CK, GK, HE, CV, and OK:** Conceived and designed the study; performed statistical analysis; supervised the study; and were responsible for the integrity of the research.

**CK, CV, and OK:** Conducted the investigation, developed the methodology, provided resources, and performed data collection.

**CK, GK, HE, CV, and OK:** Drafted the manuscript and performed review and editing.

All authors have read and approved the final manuscript and are accountable for the integrity of the study.
